# Barriers and facilitators to humanizing birth care in Tanzania: findings from semi-structured interviews with midwives and obstetricians

**DOI:** 10.1186/s12978-018-0583-7

**Published:** 2018-08-14

**Authors:** Lilian T. Mselle, Thecla W. Kohi, Justine Dol

**Affiliations:** 1 Department of Clinical Nursing, Muhimbili University of Health and Allied Sciences, Dar es Salaam, Tanzania; 2 Department of Nursing Management, Muhimbili University of Health and Allied Sciences, Dar es Salaam, Tanzania; 3 Faculty of Health, Dalhousie University, Halifax, Canada

**Keywords:** Material services, Birth care, Humanizing birth, Qualitative, Tanzania, Skilled health personnel

## Abstract

**Background:**

In Tanzania, the provision of humanized care is increasingly being emphasized in midwifery practice, yet studies regarding perceptions and practices of skilled health personnel towards the humanization of birth care are scare. Previous reviews have identified that abuse and disrespect is not limited to individuals but reflects systematic failures and deeply embedded provider attitudes and beliefs. Therefore, the current study aims to explore the perceptions and practices of skilled health personnel on humanizing birth care in Tanzania by identifying current barriers and facilitators.

**Methods:**

Semi-structured interviews were held with skilled health personnel including midwives (*n* = 6) and obstetricians (*n* = 2) working in the two district hospitals of Tanzania. Data were analyzed using thematic coding.

**Results:**

Skilled health personnel identified systematic barriers to providing humanizing birth care. Systematic barriers included lack of space and limited facilities. Institutional norms and practices prohibited family involvement during the birth process,including beliefs that limited choice of birth position as well as disrespected beliefs, traditions, and culture. Participants also acknowledged four facilitators that improve the likelihood of humanized care during childbirth in Tanzania: ongoing education of skilled health personnel on respectful maternal care, institutional norms designed for continuous clinic support during childbirth, belief in the benefit of having family become active participants, and respecting maternal wishes when appropriate.

**Conclusion:**

To move forward with humanizing the birth process in Tanzania, it will be essential that systematic barriers are addressed as well as changing the mindset of personnel towards respectful maternal care. It will be essential for the government and private hospitals to revalue their labour wards to increase the space and staff allocated to each mother to enhance family-integrated care. Additionally, in-service training as well as incorporation of respectful maternal care during pre-service training is key to changing the culture in the labour ward.

## Plain English summary

In Tanzania, many women report not experiencing respectful care from skilled health personnel when they give birth at a hospital. Previous findings suggest that there are many factors that influence whether a skilled health personnel can provide quality birth care to women including physical space and staff limitations as well as deeply embedded provider fear, attitudes and beliefs. Therefore, we decided to explore the beliefs and behaviours of nurse midwives and obstetricians in Tanzania with the goal of identifying current difficulties as well as existing enablers that allow them to provide quality care. For this study, we interviewed six nurse midwives and two obstetricians working in the two District hospitals of Tanzania. Data were analyzed using thematic coding. Only two obstetricians were interviewed because of limited number of skilled health personnel in the area. We found that participants identified barriers including physical space issues, engrained traditions within the hospitals that limited family involvement, not providing a woman the choice for the position during birth, and disregard for belief, traditions and culture of mothers. Participants also acknowledged four enablers that allow them to provide respectful maternal care during childbirth included receiving training on the need to provide respectful maternal care, hospital policies to provide continuous support and quality care, personal belief in the value of having family involved, and respecting maternal wishes during childbirth when appropriate. It will be essential for the government and private hospitals revalue their labour wards to increase the space and staff. In-hospital training and pre-service education is key to changing the culture in the labour ward.

## Background

It is well known that disrespect and abuse in maternity care is a global problem [1] with a growing emphasis on humanizing birth care to change this and improve the care women receive during childbirth [2]. According to the World Health Organization (WHO), respectful maternal care (RMC) is defined as “care organized for and provided to all women in a manner that maintains their dignity, privacy and confidentiality, ensures freedom from harm and mistreatment, and enables informed choice and continuous support during labour and childbirth” (p.3, [3]). Humanizing birth occurs when woman who are giving birth are put in the centre, with full control of the experience, working with the skilled health provider as equals to ensure evidence-based care [2]. In order for women to have a humanizing birth experience, RMC is a necessary but not sufficient requirement, yet understanding RMC can lead to a greater understanding of barriers and facilitators to fully experience humanizing birth care. Humanizing care during childbirth privileges respect towards women’s sense of dignity and autonomy without compromising their active involvement in the process of birth [2, 4].

The recognition of the need for RMC and humanizing birth care has been growing in recent years. In 2015, the WHO issued a statement on the prevention and elimination of disrespect and abuse during facility-based childbirth that called for greater action, dialogue, research and advocacy on this important public health and human rights issue [5]. Following that in 2016, The Lancet published a called to action on improving quality maternity care for every woman, everywhere [6]. Most recently, the WHO provided recommendations on intrapartum care for a positive childbirth experience where RMC was indorsed as a way to reduce maternal morbidity and mortality, improve women’s experience of childbirth, and reduce health inequalities [3]. However, in order for RMC to be implemented and to fully humanize birth care, it takes more than awareness – changes need to occur in the healthcare system as well as within individual maternal healthcare providers.

Recent systematic reviews have explored disrespect and abuse during labour and delivery from the perspective of healthcare providers and women [7–9]. These reviews highlight that abuse and disrespect is not limited to individual behaviours by midwives, but reflects systematic failures and deeply embedded provider attitudes and beliefs [7–9]. In a study with Kenyan midwives, they acknowledged that there were health system challenges, including infrastructure gaps and limited staff, that hindered their ability to implement a rights-based approach to maternity care despite a desire by providers’ to do so [10]. In a study exploring both women and midwives perspectives of disrespect during delivery care in Ethiopia, Burrowes and colleagues [11] found that while disrespect and abuse was present, the midwives reported that the abuse was unintended and reflected challenges of the healthcare system or occurred due to medical necessity.

In Tanzania,several studies highlight the importance of quality of care during childbirth [12, 13]; however, the prevalence of disrespect and abuse in a variety of healthcare and community settings is a widespread problem [12, 14–17]. For example, two qualitative studies in urban Tanzania have revealed that all participants reported experiencing or hearing about others experiences of both respectful and disrespectful or abusive care during facility based childbirth [16, 18]. However, there has been limited discussion with the midwives on their perceptions of providing RMC during childbirth in Tanzania. Women’s experience of disrespect and abuse by midwives contribute to poor access of skilled birth care in the health facility [19], denying women rights to quality maternal care [20] and contributes to maternal mortality and mortality [21].

Using participatory methods to understand and promote a culture of respectful maternal care may be key to sustainable changes [22]. Therefore, it is essential to explore the perceptions and experiences of midwives and maternal skilled health providers to understand the existing gaps and to contextualization of humanized birth care that is culturally sensitive and appropriate. The objective of this qualitative study was to describe the perceptions and practices of nurse midwivesand obstetricians (hereafter referred jointly as skilled health personnel) on humanizing birth care and barriers and facilitators to respectful maternal care in Tanzania.

## Methods

### Study Design & Setting

This study is part of a larger project exploring community and skilled health personnel perceptions and practices on humanizing birth care in Tanzania. The study was conducted in the two District hospitals in the Mwanza and Mara regions in the Lake Zone, Tanzania. Districts hospitals in Tanzania are the first referral level in the health system referral pyramid where necessary drugs, equipment, and skilled staff are supposed to be available to provide comprehensive EmOC. Further up in the health pyramid, there are regional hospitals, followed by zonal referral hospitals, and at the highest level are the national hospitals. The Lake Zone was chosen as it is one of the regions of Tanzania with the highest maternal mortality rates, with Mara having a maternal mortality ratio of 362 per 1000 births and 305 per 1000 births in Mwanza according to the 2012 census [23].

### Participants and data collection

The aim of this study was to explore the perceptions and experiences of humanizing birth care in Tanzania. Therefore, eight skilled health personnel including six midwives and two obstetricians were conveniently selected from two hospitals in the Lake Zone. Only eight skilled health personnel were interviewed, however qualitative sample size has no rule it depends on what the researcher wants to know, the purpose of the research study, and what can be done with available time and resources. It is further recommended that the minimum samples for qualitative research should be based on expected reasonable coverage of the phenomenon given the purpose of the study and interest [24]. The inclusion criteria were midwives or obstetricians working in the labour ward for a minimum of two years providing birth care and agreed to participate in the study. A midwife in charge of the labour ward (not part of the interviewed midwives) identified midwives and obstetrician who met the inclusion criteria. Throughout this paper, the term “skilled health personnel”, as defined by the 2018 WHO [25], is used to include both nurse midwives and obstetricians because of the desire not to separate out the nurse midwives and obstetricians specific findings.

The purpose of the study and principles of confidentiality were explained to participants, and thereafter, a convenient time for an interview was arranged. Semi structured interviews [26] with midwives and obstetricians who were on duty during the data collection period were conducted. A semi-structured interview guide was used focusing on skilled health personnels’perceptions and experiences of humanizing birth care (see Table 1). The guide with open-ended questions and probes used was flexible to allow the interviewer to explore issues of relevance as they emerged [27]. Interviews were conducted in aprivate, quiet room within the hospital premise at the end of the participants shift. In each interview, the participant was a major speaker and the researcher served as a guide and facilitator. The level of openness of the interviewees varied but seemed to be generally good. All interviewees agreed to the use of an audio-recorder and interviews lasted between 30–45 min. Notation of nonverbal expressions of the informants during the interview was taken during and immediately after the interview.

**Table 1:** Interview guide for Midwives and Obstetricians

SN	Interview question and probes
1	Could you please explain what kind of support do you provide to mothers during labour and delivery? (Which position did women usually assume during delivery?, who decides for delivery position?, Do women have opportunity to choose position that they would like to assume during delivery?
2	How do women’s relatives involved during labour and delivery? (Could a woman choose to be with her husband during labour?, What barriers?)
3	How do you maintain women’s privacy during labour and delivery?
4	How do you incorporate women’s cultural, spiritual and tradition believes in caring during labour and delivery?
5	What happens to the baby after delivery?

### Data analysis

Semi-structured interviews were transcribed verbatim into Kiswahili, and then translated into English by hired research assistants fluent in both languages. It was essential to translate transcripts into English to ensure access of data to non-Kiswahili speaking members of the research team. Data were analyzed using thematic coding using the English transcripts with initial codes collected and reviewed, duplicates removed, and similar codes grouped together [28, 29]. Codes and corresponding quotes were reviewed and re-labeled if necessary [30]. The semi-structured interviews yielded significantly rich data whereby no additional themes seemed to emerge, suggesting sufficient data to develop themes [30].

### Ethical consideration

This study was approved by the National Institute of Medical Research in Tanzania (Ref. no. NIMR/HQ/R.8a/Vol.IX/2143). All participants gave informed written consent to be interviewed. Participants were informed that their interviews would be recorded and agreed for their anonymous quotes to be used.

## Results

### Barriers

Four barriers were reported by participants regarding provision of humanized and respectful maternal care. This included two systematic barriers that are beyond the ability ofskilled health personnel to address on their own. These are physical space and facilities limitations, and institutional norms and practices that limit family involvement. However, it also included two individualized barriers as initiated by the skilled health personnel, including beliefs that choice of birth position should be limited as well as disrespect for belief, traditions and culture of the mother.

### Space and facility limitations

Participants reported that physical space and hospital facilities limited the ability to provide high respectful and compassionate care. The limitation of mother’s movement during labour due to limited space was reported by one of the participants:

*“It is impossible [for mothers to walk around in the labour room] given limited space in our labour room. The room has a little space for a bed and small table”.* (Skilled health personnel 1)

Physical space and facility size also limited the ability to provide privacy to mothers, which is a primary tenant to respectful maternal care.

*“[For privacy] delivery rooms have partitions. There are some partitions but the other end of the room, especially those aluminum and glass partition, do not ensure privacy of a delivering mother. Privacy does not mean only being unseen by other people when giving birth but any mother who enters the labor room expects to be alone with the midwife”.* (Skilled health personne l7)

Participants also acknowledged that the lack of space would need to be addressed if improvements were to be made to providing more humane birth care.

*“(…) our health facilities are very small. Rooms are not separated; you can easily cross from one woman to another. I think if we would like to have such things we need to make some improvements”. *(Skilled health personne l3)

*“We really do not allow them [family members] to stay with mothers during delivery because we have so many women, so you cannot allow every woman who comes in the labour room to stay with their relative, there is no place for them to stay”.* (Skilled health personnel 5)

Participants were also concerned with the limited staff that impacted the quality of maternal care provided, saying that the number of mothers who presented at the hospital for delivery exceeded the staff available during the shift to provide sufficient maternal care. The limited staff and space also limited the ability for mothers to stay long after delivery, with most mothers being discharged within 12 to 24 h after delivery.

### Institution norms and practices

Institutional norms and practices were reported as barriers that limited acceptance and encouragement of family involvement, which is a key pillar in humanizing the birth process. Participants reported that the process of humanized birth care is not fully embedded in the hospital culture. For instance, it is not common practice for family members to be involved during the birth process:

*“(…) few private hospitals in Tanzania allow this”.* (Skilled health personnel 1)

Because of this, the norm is for family members who bring the woman to the hospital for delivery to be sent home and encouraged only to visit and bring items for the mother during limited visiting hours.

*“We do not allow them [family members] to come in, once they brought in a woman  we deal with her by ourselves”.* (Skilled health personnel 1)

*“Because of our rooms is difficult to find a relative in the room all the time, we have nurses there who can provide details to relatives when they require them, but because of our rooms relatives do not stay to the end. Until the woman is delivered the relatives are outside and are not allowed to enter in the room. They will get all the details they want from nurses. Until after when the baby is born and the condition of the mother is stabilized, there is no chance for any relative to enter the room”.* (Skilled health personnel 3)

Participants also reported that they were fearful of the repercussions of having family members involved in the birth process, for themselves, their patients, and for their profession. They expressed that husbands and family members might not understand what they are doing and perceive it as abusive or harsh:

*“(…) he [husband] might see the way we are handling his wife as inappropriate and react accordingly”.* (Skilled health personnel 4)

It may also lead to breach of confidentiality of their clients’ information:

*“(…) besides, when the relatives are too involved at this critical time, they may divulge sensitive medical information to the mother and share the medical records publicly by passing it to other people (…)”.* (Skilled health personnel 8)

In terms of the fear related to their profession, one skilled health personnel explained that despite the recognition that family members should be involved, they still feared a negative outcome.

*“Panic can emerge as the result of the spouse beholding the critical health condition of the woman in labour, where the mother is experiencing excessive bleed or the midwife is pressing the woman’s abdomen to push the baby out. These situations may be perceived by the spouse as coercive or abusive. This perception may cause the spouse to consider legal action against the hospital. So, yeah, much as everyone has rights, we strive to avoid such problems by encouraging minimal spouse involvement.”* (Skilled health personnel 8)

### Beliefs that choice of birth position should be limited

It was learnt from the participants that they preferred women to deliver in the lithotomy position as it makes it easier for the skilled health personnel to assist with delivery and helps the baby deliver smoothly.

*“Lithotomy position for pregnant woman is better because it will be easy to see as the baby protrudes through the woman’s vulva. (…) it is also good for the midwife to check the cervix of the mother who is about to give birth compared to when the mother is in the lateral position. In my view, the lithotomy position is better because less time will be spent for child delivery”*. (Skilled health personnel 5)

Another participant also shared that lithotomy is a professionally sanctioned position:

*“[The lithotomic position] is the professionally sanctioned standard position (…) it is the most accommodating in that it gives the mother considerable pushing power and allows the mother to grab her legs when [pushing], which is the safest way*”. (Skilled health personnel 8)

*“(…) what I was taught is that that is [lithotomy] a good position and it helps a mother to give birth well and exactly depending to how a baby lies. You can help her well and you can receive the baby in good condition and it makes the mother to be more comfortable. (…) I have also read that lithotomy position is best and delivery can be conducted easily”.*(Skilled health personnel 3)

Skilled health personnel expressed that because of this preference, they often told the woman to get into this position during delivery, rather than allowing her to decide the position she would prefer to use when giving birth:

*“You are [midwife] the one who tells the woman which position she should lie for easy delivery because other women it is their first delivery so they do not know”. *(Skilled health personnel 4)

*“We [midwives] have experience with that position, the one which she lies on her back”. * (Skilled health personnel 3)

*“Many women give birth lying on their backs. Many say that they feel more comfortable when they deliver they get strength. (…) we normally don’t ask but we instruct them how to lie”.* (Skilled health personnel 6)

*“[Some mothers prefer] to sit, others prefer a delivering posture the same as they are in the toilet. But others prefer to lie in sideways. I think that, on the side of the woman, the best posture is that which she feels comfortable. As a service provider, I prefer the lithotomy position, especially when we want to maintain the mother’s cleanliness. This allows me to control a mother when assisting her during delivery. So it is a two-sided perspective”.* (Skilled health personnel 7)

### Disrespect for traditions and culture

Participants reported disrespect or disregard for the traditions and culture of the women who come to give birth. They acknowledged that they do not recognize the tradition norms but instead encourage the westernized, evidence-based medicine that is provided in hospital. Participants explained their hesitation with the use of herbs used by women to facilitate labour:

*“Our perspective is that herbs are not scientifically tested. Although the herbs help in accelerating contraction, they have side effects. (…) we don't know exactly its composition. Actually, the issues of tradition and customs is not given due weight. I personally do not take it as a serious issue. I work as I was trained. (…) we may be performing our duties as Europeans. I take the medical training that I received is of European norms and principles. So, we take that understanding expecting mothers who has come to the hospital for treatment or childbirth has left her traditions and customs at home. She has agreed with European norms. She may perform her traditions and customs when she goes back home. I have not asked any mother about her observance regarding childbirth according to her tribe”.* (Skilled health personnel 7)

Participants also explained how their previous experience with women using traditional medicine had done more harm than good for the mother and baby, and thus they devalued their importance. One skilled health personnel shared the story about the dangers of using traditional medicine:

*“Most women use herbs to facilitate uterine contractions. (…) we need contractions but with normal required intensity and frequency. (…) most of the expecting mothers use herbs before coming to the hospital and they end up with fetal distress, because she may have good contractions but with an unopened cervix. In this situation, contractions do not go with labor progress (…)”.* (Skilled health personnel 7)

### Facilitators

Despite the barriers described for humanizing birth care, participants also acknowledged several facilitators that are currently in place that could improve the provision of respectful maternal care during childbirth. This included systematic facilitators such as the ongoing education of skilled health personnel on the need to provide respectful maternal care and institutional norms designed for continuous clinic support during childbirth. Additionally, personal factors among the skilled health personnel that encourage respectful maternal care include the belief in benefit of having family to become active participants as well as respecting maternal wishes during childbirth when appropriate and safe.

### Ongoing education of skilled health personnel on respectful maternal care

Despite the barriers and institutionalized barriers, skilled health personnel described receiving education on key behaviours that reflected respectful maternal care. Skilled health personnel reported receiving training on different delivery positions as well as the need to respect the rights of the mother during the delivery:

*“(…) even in class we were told the mom [can] choose [the position] how to push”.* (Skilled health personnel 1)

*“As we were taught, we have to allow the woman to assume the position which she is comfortable to give birth, although most of time we advices them to lie with their back. But if she wants to squat, you have to allow her”.* (Skilled health personnel 3)

*“(…) we were educated about importance of maintaining privacy, ensuring the woman in labour is constantly supported physically and emotionally by providing psychological support and managing pain (…)”.* (Skilled health personnel 1)

### Institutional norms designed for continuous clinic support during childbirth

Participants provided insight into current practices within the hospital that involve providing continuous support for mothers during childbirth. While not always provided as evidenced through above barriers, participants noted that throughout the birth process, the skilled health personnel attempted their best to provide continuous care:

*“(…) women are helped from the first stage until the fourth stage of labour and we stay with her until she is discharged and she becomes free with her relatives”. *(Skilled health personnel 1)

*“During a typical delivery, the doctors, while they were not always present during every uncomplicated delivery, they were close by and active in complicated deliveries or during operations”.* (Skilled health personnel 4)

The participants explained the delivery process for mothers who come for birth. They commonly assess the woman during admission determining what stage of labour she is in:

*“(…) when a woman arrives at the reception, we always receive her quickly and do quick assessment, we prioritize women because they arrive many at once. (…), you have to check what kind of women you have because some are in true labour pain and close to deliver or sometimes we like to ask those who have urge to push”. *(Skilled health personnel 1)

If she is in the first stage of labour, she is placed in the observation room and re-assessed every four hours or until she notifies a skilled health personnel a change in her labour status. If she is in second stage of labour, they bring her immediately to the labour room where she is continuously monitored until delivery:

*“When the mother arrives with labour pain, I receive her and start physical examinations. I check her to ensure her blood pressure is not high which can cause problems during delivery. After doing that then I do abdominal examinations and PV examination to asses if the cervix is open or not and if yes by how much; so that to determine the progress of labour or she will be taken to labour room directly, because others arrive already in second stage (…). After completing examinations I then check her health status; if she has tested or not. I test her if she hasn’t been tested to know if she has acquired infection or not (…)”.* (Skilled health personnel 4)

The privacy is attempted to be maintained during delivery:

*“(…) here at our facility we use curtains to maintain privacy. If a woman wants to give birth, we must make sure all curtains are set. In our labour ward, there are partitions so we close the curtains very well”.* (Skilled health personnel 2)

Once the baby is delivered, they assess the baby and transfer the mother and her baby to the postnatal ward for recovery.

*“[After the baby is born] we look up at the child's scoring, appearance (…) we also look at how the baby is crying and then we wrap up the baby. Thereafter we weight the child on a scale. After these procedures, we instantaneously carry the baby to a parched area and immediately put her to breastfeeding”.*(Skilled health personnel 7)

Thus, current practices in the labour ward attempt to offer continuous support to mothers and their babies, despite the barriers faced by skilled health personnel. Providing a dehumanized birth experience was not expressed as a desire by any skilled health personnel.

### Belief in benefit of having family to become active participants

Despite space limitations in the labour wards, participants acknowledged the benefit to the woman to have family members be active participants during the delivery process. Skilled health personnel gave the following justifications when explaining the benefits to having family members involved:

*“(…) if the partner is present from the beginning of labour up to delivery, then he will understand what the mother has been through and hence be able to help her while at home”.* (Skilled health personnel 4)

*“(…) a partner being present during labor is to help the expectant mother emotionally and to encourage the mother to push. (…) there are few midwives and most of the time they are outnumbered by the expectant mothers. There may be up to seven expectant mothers on beds with only one midwife making it difficult for her to work on time and with efficiency”.* (Skilled health personnel 3)

*“(…) normally they come with their mothers. I think her mother should be there. Because she understood, she is able to guide her (…)”.* (Skilled health personnel 6)

### Respecting maternal wishes when appropriate

As skilled health personnel were taught behaviours related to respectful care, they reported that they would respect maternal wishes when it was appropriate. Participants explained that they might not understand some of the decisions that women made. However, if the decision did not limit their ability to provide quality and safe care to the mother and the baby, they would allow the mothers’ wishes to be followed. A skilled health personnel explained that despite suggesting the lithotomy position, if a mother wants to give birth in another position, she obliges:

*“(…) if she tells us her best position then we have to support her in that position for her to give birth comfortably”.* (Skilled health personnel 3)

Likewise, another participant explained how they dealt with traditional beliefs and practices:

*“In my perspective, we are not that thoughtful of the traditional practices but we are always accommodative of the mother and family wishes, if there are any. Whether it’s the longing to pack up the placenta back home or this and that, one should simply let the mother fulfil her wish”. *(Skilled health personnel 7)

## Discussion and implications for midwifery and health policies

Overall, skilled health personnel identified several barriers that limited their ability to provide humanized birth care including systematic issues, including physical space and institutional norms and practices as well as personal barriers, including beliefs that choice of birth position should be limited and disrespect for tradition. Nevertheless, several facilitators were identified that are currently in place that can move the process of humanizing birth forward, including ongoing education of midwives on respectful maternal care, institutional norms designed for continuous clinic support during childbirth, belief in the benefit of having family become active participants, and respecting maternal wishes when appropriate.

The current findings are consistent with the recent systematic review by Bradley et al. [8] that highlighted that abuse and disrespect is not limited to individual skilled health personnel but reflects systematic failures and deeply embedded provider attitudes and beliefs [7, 8]. In Bradley et al.’s conceptual framework based on their meta-analysis of women’s perceptions of intrapartum care in Sub-Saharan Africa, macro-level factors (colonial legacy, structural inequality, and health system policy & drivers) and meso-level factors (medicalization of birth, midwifery history and training, hierarchical and institution-centred, work environment and resources, poverty and inequality, gender inequality/status of women) influence and shape the inter-personal dynamics happening at a day-to-day micro-level for skilled health personnel [8]. For instance, in our study, we found that skilled health personnel desired women to accept Western norms around birthing position, echoing the colonial legacy and the ongoing medicalization of birth in these countries, reflecting macro- and meso-level factors. Challenges remain in shifting the attitudes and behaviours of all skilled health personnel to embrace evidence-based practices to enhance the humanizing birth experience, even if it varies from Western-based medicine.

To move forward with humanizing the birth process, there is a need to address the barriers and enhance the facilitators as identified by the skilled health personnel. First, it will be essential that individual level issues are addressed. Individual level barriers included currently held beliefs that choice of birth position should be limited and ongoing disrespect for tradition. Yet skilled health personnel also identified corresponding facilitators, including holding the belief in the benefit of having family become active participants and respecting maternal wishes when appropriate. It is clear from our findings that there is a desire to offer respectful maternal care and a humanizing child birth experience, yet it has not fully emerged in practice. There exists some discord around what skilled health personnel think and desire to happen, versus what is actually happening in the unit. For example, some participants reported that privacy was consistently provided to mothers, yet others reported that due to institutional barriers, this was not always provided. This is not unique to our study, as Warren et al. [31] found that both respect and disrespect were considered common among midwives in Mali.

It is essential to change the mindset of skilled health personnel towards humanizing birth care, particularly around the delivery position and respecting tradition if it does not impact safety and quality of care. One of the facilitator mentioned was that skilled health personnel are taught about different delivery positions and that women should be allowed to choose which position they would like to assume during delivery, yet the culture on the labour wards does not encourage this in practice. The issues of skilled health personnel exercising power and control, particularly through controlling the position a women assumes and limiting movement, to assert their professional identity, and to take charge of both women and the birth process has been acknowledged elsewhere [8]. This behaviour is thought to be related to the need for skilled health personnel to maintain a particular social status compared to the women (the skill health personnel’s role and social distance and ‘othering’) [8]. In a systematic mapping by Filby et al. [7] of midwife-identified barriers in providing quality care, they identified professional, social, and economic barriers, which resulted in burn out and moral distress for midwives when not addressed. Therefore, it will be important to target their concern related to maintaining their professional identity as skilled health personnel as well as ensure they are offered the professional and social support needed to provide humanizing birth care to mothers.

However, the physical space within the current labour wards significantly limits skill health personnel’s ability to provide a humanizing birth experience. If there is limited space and a need to enhance women’s privacy, it is impossible to allow family members to join the mother during the delivery process despite the acknowledgement by participants that this would be beneficial. Additionally, the limited staff also hinders the ability to provide quality care, despite the desire to provide continuous support and quality care. The issue of space limitations related to humanizing birth care due to poor infrastructure or the health care system is not unique to Tanzania [4, 10, 11, 32] and remains a larger issue that needs to be addressed moving forward. In order to provide a humanizing birth experience in Tanzania and beyond, consideration of changes to the physical space is warranted related to systematic issues.

Education to individual skilled health personnel alone is not sufficient without education occurring also at the administration and policy level, where the power to influence change at the systematic level can occur. Without this, even skilled health personnel who want to provide a humanizing birth experience to mothers are limited by space and institutional norms that hinder these opportunities [4]. As seen in the *Heshima* project in Kenya, when providing education to both providers and managers related to respectful maternal care, there was an increase in knowledge and practices among providers for understanding client rights and client-centered care [10]. However, the authors noted that challenges remained due to peer influence, whereby the existing work culture limited the ability of healthcare providers to change behaviour, even if they so desired [10]. To encourage humanizing birth care, more than just individual factors related to skilled health personnel need to be addressed but includes systematic issues that need to be considered moving forward. Interventions targeting both skilled health personnel and administrations have shown some success in reducing disrespect and abuse in Tanzania [15, 33], yet further research is needed to fully understand how women’s values, belief and providing respect and autonomy during the birth process can be achieved. The WHO identifies that in order for RMC to occur, there needs to be systematic resources in place, including trained and skilled health personnel, adequate physical infrastructure, and accountability within the ward [3].

### Limitations

While this study was conducted with rigor and provides important insights into the humanizing birth care barriers and facilitators in Tanzania, there are some limitations that should be acknowledged. First, the study was conducted at two district hospitals in two regions of Tanzania where participants were employed. Participants may be unlikely to report any disrespectful care that either they or their colleagues engaged in due to fear of repercussion. However, all attempts were made to ensure confidentiality and participants were informed that their responses would not be shared with their employers. While there is always the risk of social acceptability bias, that is that the health care providers would try to minimize their lack of respectful care when speaking to other health care providers, this may have been minimized by the fact that the nurse-midwives who conducted the interviews did not work in the hospitals where the research was conducted and that they had extensive experience in conducting health research and trying to probe for the interviewee’s real perspectives. Furthermore, despite this risk, participants identified several barriers that limited respectful maternal care, suggesting that participants were honest in their responses. Nevertheless, data from interviews should be interpreted with some caution as conducting interview at the health facility there is a risk of ‘courtesy bias’, or participants providing what they believe are acceptable responses rather than their own opinions.

Another limitation is that this study included only two obstetricians because of a low number of skilled health personnel in this category available in the district hospitals in this area of study. Nevertheless, it was important to capture the perspective of both obstetrician and nurse midwives as both categories of skilled health personnel provide ongoing care to mothers within these hospitals. As both nurse midwives and obstetricians are involved in providing birthing care at these District hospitals, our sample reflects the skilled health personnel involved in providing humanizing birth care and their interpretation of facilitators and barriers.

A final limitation is that analysis of interviews was completed in English from translated transcripts, which may have impacted the analysis. However, transcripts were verified by research team members fluent in Kiswahili to ensure adequate translations and all codes and themes were discussed amongst the researchers who were able to review the original transcripts. Additionally, after every interview, researchers had opportunity to listen to the audio recorded interviews, reflecting on the interview sessions and information gathered in the reflection sessions, which were used during analysis to complement interpretation of themes.

## Conclusion

The findings of our study provide valuable insight into the current perceptions and practices by skilled health personnelon humanizing birth care in Tanzania. Several barriers were identified in providing respectful maternal care including systematic and personal barriers, yet several facilitators existed that could be encouraged and enhanced to address the barriers. To humanize the birth process in Tanzania, it will be essential for the government and private hospitals to revalue their labour wards to increase the space and staff allocated to each mother to enhance family-integrated care. Additionally, in-service training as well as incorporation of respectful maternal care during skilled health personnel training is key to changing the culture in the labour ward.
